# Breast cancer care in northern Ethiopia – cross-sectional analysis

**DOI:** 10.1186/s12885-019-5612-6

**Published:** 2019-04-25

**Authors:** Biniyam Tefera Deressa, Nikola Cihoric, Eugenia Vlaskou Badra, Alexandros Tsikkinis, Daniel Rauch

**Affiliations:** 1 University of Gondar, Gondar, Ethiopia; 2 Department of Radiation Oncology, Inselspital, Bern University Hospital, University of Bern, Bern, Switzerland; 3 Regional Hospital Thun, Thun, Switzerland; 4 Department of Medical Oncology, Inselspital, Bern University Hospital, University of Bern, Bern, Switzerland

**Keywords:** Breast cancer, University of Gondar Hospital, Northern Ethiopia

## Abstract

**Background:**

In Ethiopia, the incidence of new cases of breast cancer is currently increasing resulting to high rates of morbidity and mortality. Breast cancer is by far the most common cancer accounting for more than one out of three cancer cases in women and one out of every five in the general population. The study was conducted in University of Gondar Hospital cancer center, located in the North-West Ethiopia; to evaluate the clino-pathologic characteristics of breast cancer and care provided for patients.

**Methods:**

All biopsy proven breast cancer patients treated between 2016 and 2017, were identified and information regarding histology, stage, therapeutic procedure and follow up was retrospectively collected from their individual medical records and descriptive analysis was done.

**Results:**

Among 82 patients treated, 67 (82%) were women and 15 (18%) were men. The median age at the time of diagnosis was 45 years (25–82 years). Operation was performed for 56 (68%) patients. The predominant histology was ductal carcinoma in 61 patients (74%), followed by breast carcinoma of No Special Type (NST) in 17 (21%). The late presentation of the patients and the advanced stage at the time of presentation was observed in most of the patients. Chemotherapy was administered in 79 (96%) patients. Radiotherapy was not available in the hospital.

**Conclusion:**

Breast cancer incidence is rising and becoming a major public health problem in Northern Ethiopia. Breast cancer care in northern-Ethiopia is limited in terms of both pathology, imaging and the offered treatment modalities, which need to be improved.

## Background

Breast cancer is the most frequently occurring cancer in women, with approximately 1.7 million new cases and nearly 522,000 related deaths worldwide in 2012 [1]. In Ethiopia the incidence of new cases of breast cancer is currently increasing and has become one of the most common cancer types causing high rates of morbidity and mortality [2–4]. According to the study by Memirie et al. breast cancer is the most common cancer, constituting 33% of the cancers in women and 23% of all cancers in Ethiopia. The Estimated national Age Standardized Incidence rate for women was about 43 per 100,000 population [5].

The recently reported, first data from the Addis Ababa City Population based cancer registry shows that breast cancer is the most common cancer in women and one of the top Ten cancers in men. Breast cancer incidence has shown a steady yearly growth at the Tikur Anbessa Specialized Hospital, the only hospital in the country where cancer patients have access to oncology care [6, 7]. Furthermore, the majority of patients have at the time of presentation advanced disease with 67% presented with locally advanced and 25% metastatic disease [8]. The combination of all the aforementioned factors leads to unsatisfactory treatment outcomes [8, 9].

The Ethiopian government has recognized this as a major problem and has set a priority the advancement of cancer care in the country. In 2015 the Ethiopian healthcare authorities developed a cancer control plan, which is focusing on the prevention as well as the general improvement of cancer care in the country [10, 11]. One of the most significant steps taken was the establishment of 6 specialized cancer centers at the teaching university hospitals of Gondar, Hawassa, Mekelle, Jimma, Haramaya and St. Paul’s Hospital [12].

### Cancer care at the university hospital of Gondar

The University of Gondar Hospital is a tertiary teaching hospital, located in the North-West Ethiopia providing healthcare for a population of approximately 5,000,000 people. The hospital has around 600 beds with 400,000 patients treated annually. The hospital has more than sixty years’ experience in teaching, research, health care delivery and other community services employing 250 general practitioners, 20 internal medicine specialists, 14 surgeons, 12 pediatricians, 7 gynecologists and more than 30 other medical specialists. The University Hospital also has more than Ten years of experience and participation in clinical trials focusing mainly on infectious diseases such as leishmania and HIV infection [13, 14]. Until recently however, specialized and comprehensive cancer care was not offered at the hospital, with just selected cases being operated on and with the bulk of diagnostic and interventional procedures being referred to the Tikur Anbessa Specialized Hospital located 900 km from Gondar.

In January 2015 the Gondar University Hospital together with the BEZA Association of Switzerland, established the first cancer treatment center in the region [15]. The center includes Ten beds for outpatients as well as an in-patient care unit with Eight trained nurses, Two general practitioners and One senior oncology resident from the Tikur Anbessa Hospital on a monthly rotation. Furthermore, a senior oncologist from Switzerland regularly visits the center. One of the major achievement of the center was the establishment of a regular multidisciplinary tumor board consisting of a group of medical professionals including a pathologist, a radiologist, a surgeon and the attending physician.

The histopathological evaluation is limited on the morphology, with immunohistochemistry and other molecular tests currently unavailable. The department of radiology is equipped with a CT-Scanner, an X-ray machine and Ultrasound devices. The University of Gondar Hospital cancer center provides medical oncology service such as the administration of chemotherapy and hormone therapy for several tumor entities. Radiotherapy is still not available at the center and those patients requiring radiotherapy are referred to Tikur Anbessa Hospital in Addis Ababa.

Breast cancer is one of the most common cancer entities treated at the University of Gondar Hospital, therefore a retrospective evaluation was conducted to detect patterns of care and evaluate early results [16].

## Methods

The medical record review was done by physicians working in the center. First, we have retrieved medical records of all breast cancer patients. Secondly, we have reviewed records and identify variables that can be extracted from the record. The completeness of the record was ascertained by the primary investigator. Data was collected on tumor stage, lymph node status, and presence of metastasis. The staging in Gondar University Hospital is based on the presence or absence of symptoms and clinical evidence of the disease. In addition to the clinical evaluation, every patient with locally advanced disease was evaluated at least with a chest x-ray and abdominal ultrasound. Patients were staged according to the TNM staging system of the American Joint Committee on Cancer (AJCC), seventh edition [17]. The date of morphological confirmation of breast cancer was considered as date of diagnosis. Pretreatment evaluation included evaluation of complete blood count, renal function tests and liver function tests. In addition to staging data, we have collected data on therapeutic procedures and follow-up. The study was conducted according to the local ethical guidelines.

## Result

From December 2016 to November 2017, 82 patient were treated at the center. Sixty-seven patients (n:67, 82%) were females and 15 male (n:15, 18%). The median age for all patients was 45 years (range from 25 to 82 years). About 47% of the patients were 40 years old or less, 27% between 40 to 50 years of age and the rest 26% were above age 50. Of the 67 female patients 38 patients (57%) were premenopausal and 29 patients (43%) were post-menopausal. The 15 male patients were generally older than their women counterparts with a median age of 65 (range 40 to 82 years). The age distribution is depicted in Table 1.

**Table 1:** Clinical and pathological characteristics of the patients

Characteristics	Number	Proportion
Total Population	82	100%
Duration of symptoms in months		
≤ 4	8	10%
5–8	20	24%
9–16	28	34%
17–24	15	18%
≥ 25	11	13%
Menopausal Status (Women Only)		
Pre-menopausal	38	57%
Post-menopausal	29	43%
Main presenting Symptom		
Breast pain	1	1%
Breast Swelling	65	79%
Pain outside breast	1	1%
Ulceration	15	18%
Lateralization		
Right Breast	48	59%
Left Breast	32	39%
Bilateral	2	2%
Type of Surgery performed		
Surgery was not performed	26	32%
Modified Radical Mastectomy	51	62%
Simple Mastectomy	2	2%
Palliative Mastectomy	3	4%
Histology		
Ductal Carcinoma	61	74%
Breast Cancer NST	17	21%
DCIS	1	1%
Lobular Carcinoma	1	1%
Mucinous Carcinoma	1	1%
Papillary Carcinoma	1	1%
Grade of Differentiation		
G-1	14	17%
G-2	30	37%
G-3	26	32%
Not reported	12	15%
First-line chemotherapy Given		
AC	50	61%
FAC	14	17%
AC + taxol	13	16%
Chemotherapy not started	3	4%
CAF	1	1%
CMF	1	1%
Hormonal Therapy		
Anastrazole	6	7%
No hormonal therapy	22	27%
On Chemotherapy	36	44%
Tamoxifen	18	22%

The main presenting symptoms at presentation was a longstanding breast mass in 65 patients (79%) and breast ulceration in 15 patients (18%). Only one patient reported breast pain as initial symptom and one patient presented with pain outside of the breast. In 48 patients (59%) the lesion was in the right breast, in 32 patients (39%) in the left breast and 2 patients (2%) had bilateral breast lesions. The median duration from first symptom to the time of diagnosis was twelve months (range 3 weeks to 5 year) (Table 1).

All of the patients had a metastatic work up at least with Chest X-Ray and abdominal Ultrasound. One of the 82 patients had a mammography as initial work-up and 23 (28%) of the patients had a breast ultrasound.

Fifty-six (68%) patients received an operation, the most common surgical procedure was modified radical mastectomy (*n* = 51), followed by simple mastectomy (*n* = 2) and palliative mastectomy (*n* = 3). Surgery was omitted in 26 (32%) patients due to the advanced stage of the disease with metastasis at presentation (Table 1)

The predominant histology was ductal carcinoma in 61 patients (74%), followed by breast carcinoma of No Special Type (NST) in 17 (21%) patients. Ductal Carcinoma In-Situ (DCIS), lobular carcinoma, mucinous carcinoma and papillary carcinoma were found in one patients each. The grade of differentiation was well differentiated in 14 (17%), moderately differentiated in 30 (37%), poorly differentiated in 26(32%) and unknown in 12 (15%) patients. Surgical margin status was reported for 29 patients, with 23 patients having surgical margins free of tumor and 6 infiltrated by the tumor. Since the immunohistochemistry tests are not fully installed in the hospital, only 2 patients had tests for hormonal status (expression of estrogen and progesterone receptors) to which both were found to be positive (Table 1).

The stage at presentation was locally advanced or metastatic for most of the patients. Distribution according to stage at presentation is plotted on Table 1.

All but three patients (96%) received chemotherapy. Of those 50 patients (61%) received Adriamycin and Cyclophosphamide (AC-regimen), 14 patients (17%) Fluorouracil, Adriamycin and Cyclophosphamide (FAC-regimen), and 13 patients (16%) Adriamycin and Cyclophosphamide plus Paclitaxel (AC-Taxol regimen). Cyclophosphamide, Methotrexate and Fluorouracil (CMF-regimen) and Fluorouracil, Adriamycin and Cyclophosphamide (FAC-regimen) were each administered in one patient each. Among the patients who completed a full course of chemotherapy, 24 patients received empiric hormonal therapy and 22 patients did not receive any hormonal treatment. The rest of the patients were on chemotherapy treatment. The summary of the clinical and pathologic characteristics of patients treated in University of Gondar hospital cancer center is shown in Table 1.

## Discussion

Most of the patients treated at the Gondar university hospital cancer center were young with almost half of them being less than 40 years old. This finding is similar to the reports of previous breast cancer studies completed in Ethiopia [7, 9, 18–20]. This however, is different from the current trend in western countries where only less than 7% of the patients diagnosed are younger than 40 years of age [21, 22]. The reason for the high number of younger patients in Ethiopia is currently unclear. A potential explanation for this is possibly the demographics of Ethiopia and the high percentage of younger individuals in the population of the country [18, 23]. Indeed, this is a logical explanation since reports in our findings and other previous studies were not age adjusted. Nevertheless, several studies have shown that tumors arising in younger patients are more aggressive and those patients are at a higher risk of relapse and death not only due to their age but also due to the unique tumor biology [24–26]. Therefore, further research is required to identify or exclude potential underlying genetic or environmental factors that would render women in Ethiopia more prone to develop the disease at a young age.

Another finding of our study is the high proportion of male breast cancer, which accounts for 18% of the total patient population during a calendar year. Unlike breast cancer in women, the median age of breast cancer in men in our findings is similar to the median age reported in western countries [27]. A similar percentage of male breast cancer patients was reported from this hospital in 2016, with breast cancer being among the top 10 cancers diagnosed in men [16]. The rarity of the disease worldwide which accounts for less than 1% makes this finding interesting [27]. The reason/s for the higher incidence of male breast cancer is not clear. A possible explanation for this are unique cultural factors and/or economic reasons that would explain why men seek healthcare in rural Ethiopia more often than women do [28]. However, reports from the Addis Ababa city population based cancer registry, which includes mostly urban population shows a higher proportion of male breast cancer cases, of about 7% and still one of the top 10 cancers in men [2]. In conclusion, other possible environmental or genetic factors might be implicated in the high incidence of male breast cancer in Ethiopia. Considering the fact that the higher percentage of male breast cancers is encountered in other Sub-Saharan countries as well as blacks living in the United States where the higher male-to-female ratio is also seen, it is possible that a yet unknown genetic component is potentially instrumental for this phenomenon [29]. Since male breast cancer biology or treatment is not well understood due to its rarity, Ethiopia could be one of the study sites for future clinical trials to boost our knowledge on the topic.

The other findings of our study is the late presentation of the patients and the advanced stage at the time of presentation. About 85% of the cases were diagnosed at a stage III and IV. This finding was similar with previous breast cancer studies from Ethiopia and other African countries such as Tanzania, Uganda and Egypt [7, 9, 30–32]. The absence of screening combined with the low rate of cancer awareness and unavailability of multi-modality treatment may be the reason for the late presentation and diagnosis at an advanced disease stage. It is obvious that the late stage at presentation negatively affects the breast cancer survival [33]. As it was clearly shown on the recent publication of Weiner et al., the average survival probability of metastatic breast cancer in Ethiopia was about 12 months which was significantly lower than western countries [34]. Together with lack of standard therapy, late stage at presentation could be one of the reasons for the higher mortality rates attributed to breast cancer patients [34]. Although the incidence rate of breast cancer is lower in Africa than in high income countries, there is a disproportionately higher number of mortality [35, 36]. The age-standardized breast cancer incidence rate is 70.08 in Europe and 28.66 in Africa, while the mortality rate is about 15.93 in Europe and 13.72 in Africa [37, 38]. The five-year survival rates is around 80% in the high-income countries and 40% in the low-income countries [39]. The higher mortality rates for breast cancer in Africa and other LMICs, is due to the rising incidence as well as late-stage diagnoses and limited access to treatment [40].

The major limitation of the present study is its small sample size. The total number of patients treated in the center could be relatively smaller compared to the total population in the area. This could be due to lack of awareness of the population, seeking of alternative traditional treatments or limitation of knowledge and diagnostics facilities from health care providers. The other reasons could be due to lack of information and thus, less referral of the diagnosed or surgically treated patients to the center as it was newly established.

We believe that the strength of the study could be increased if it had been possible to show the outcome of the breast cancer patients in the hospital in terms of overall survival or progression free survival. Our reason for not including survival analysis was that, the medical record of patients existing during the study period didn’t have detailed address or phone number of the patients which makes it difficult to get vital status. In spite of these limitations, our first report on breast cancer clinical, pathologic and treatment pattern may have an impact in sensitizing third parties and thus ultimately improving the healthcare offered. It could also be the base for future larger studies.

## Conclusion

Breast cancer incidence is rising and becoming a major public health problem in Northern Ethiopia. Breast cancer care in northern-Ethiopia is limited in terms of both pathology, imaging and the offered treatment modalities, which need to be improved. Furthermore, Ethiopia could potentially become one of the major research areas in terms of younger individuals and men with diagnosed breast cancer.

## Abbreviations

AC-regimen: Adriamycin and Cyclophosphamide
AC-Taxol regimen: Adriamycin and Cyclophosphamide plus Paclitaxel
AJCC: American Joint Committee on Cancer
CMF-regimen: Cyclophosphamide, Methotrexate and Fluorouracil
DCIS: Ductal Carcinoma In-Situ
FAC-regimen: Fluorouracil, Adriamycin and Cyclophosphamide
HIV: Human Immuno-deficiency Virus
LMICs: Low- and Middle-Income Countries
NST: No Special Type
TNM: Tumor, Node, Metastasis
